# Impact of Air Pollution on COPD; Underlying Mechanisms

**Published:** 2017

**Authors:** Hasan Bayram

**Affiliations:** 1 University of Gaziantep, Turkey;; 2 Koc University, Istanbul, Turkey.

Epidemiological studies have demonstrated that there is an association between increases in air pollution and cardiopulmonary mortality and morbidity. Multi-centre studies in North America and Western European countries reported that an increase in the levels of particulate matter (PM), ozone, nitrogen oxides (NOx) and sulphur dioxide (SO2) leads to increases in prevalence, emergency room visits and hospitalization due to chronic respiratory diseases such as asthma and chronic obstructive pulmonary disease (COPD). In our recent studies, we investigated mechanisms underlying pollutants-induced effects on airways of COPD patients. We cultured airway epithelial cell lines, and primary bronchial epithelial cells (BECs) from non-smokers, smokers with and without COPD, and investigated effects of diesel exhaust particles (DEP), which constitute the major fraction of particulate air pollution, on inflammatory mediator expression and viability and proliferation of these cells. Although DEP generally induced A549 alveolar epithelial cell viability, on contrary these particles inhibited the viability of BEAS-2B bronchial epithelial cells and primary BECs. Looking for the underlying mechanisms, we observed that DEP suppressed apoptosis of A549 cells, while inducing the apoptosis of BEAS-2B and primary BECs. Our real time-polymerase chain reaction (RT-PCR) studies demonstrated that DEP modulate mRNA expression of proteins regulating cell proliferation and apoptosis. Furthermore, DEP affected protein release and mRNA expression of inflammatory cytokines such as interleukin (IL)-8 and granulocyte macrophage colony stimulating factor (GM-CSF). This effect was modulated by *N*-acteylcysteine, and the inhibitors of cell signalling pathways. Our findings suggest that DEP may play a role in the pathogenesis of chronic pulmonary diseases such as COPD, by modifying viability, apoptosis, cytokine release and cell proliferation and apoptosis regulating proteins of BECs and alveolar epithelial cells.

